# The effect of aromatherapy with lavender essential oil on the working memory of women with multiple sclerosis

**DOI:** 10.25122/jml-2020-0115

**Published:** 2021

**Authors:** Sara Mohammad Rezaie, Maryam Shahabinejad, Marzeyeh Loripoor, Ahmad Reza Sayadi

**Affiliations:** 1. Internal Surgery Nursing, Rafsanjan University of Medical Sciences, Rafsanjan, Iran; 2. Department of Medical Surgical Nursing, Rafsanjan University of Medical Sciences, Rafsanjan, Iran; 3. Department of Midwifery, Rafsanjan University of Medical Sciences, Rafsanjan, Iran; 4. Department of Psychiatric Nursing, Rafsanjan University of Medical Sciences, Rafsanjan, Iran

**Keywords:** aromatherapy, lavender, working memory, multiple sclerosis, women

## Abstract

Working memory, one of the cognitive components, may be impaired in patients with multiple sclerosis. Accordingly, this study aims to determine the effects of aromatherapy with lavender essential oil on the working memory of women with multiple sclerosis (MS). In this clinical trial, 60 women with multiple sclerosis were selected using the sampling method from patients referred to the MS Clinic of Rafsanjan. Based on the inclusion and exclusion criteria, the participants were randomly divided into intervention and placebo groups. In addition, the working memory test developed by Daneman and Carpenter was used to evaluate the participants’ working memory before the intervention and the day after the last intervention. The collected data were analyzed using SPSS Statistics version 18.0. According to intragroup comparison results and based on the paired t-test, the mean score of the working memory before the intervention in the intervention group was 82.77±6.87, which increased to 87.64±5.57 after the intervention (P<0.001). The average working memory score of the placebo group was 80.30±11.09 and 82.09±11.31 before and after the intervention, respectively, which did not have a statistically significant difference (P=0.154). Based on findings from the independent t-test, the mean scores of working memory had a statistically significant difference between the intervention and placebo groups after the intervention (P=0.02). According to the results from this study, aromatherapy with lavender essential oil improved working memory in women with multiple sclerosis.

## Introduction

Multiple Sclerosis (MS) is an autoimmune and chronic disease of the central nervous system (CNS), which destroys myelin and damages axons at different levels [1]. The causes of MS are still largely unknown, and there are currently no known factors that can help prevent the disease. However, genetic and environmental risk factors can contribute to the disease [2]. It is estimated that 2.3 million people worldwide suffer from the disease [3]. According to epidemiological studies, the prevalence of MS in Iran, especially among women, is increasing rapidly [4], with women being twice as likely as men to be affected by the disease [5]. Working memory is a type of memory, which is impaired in people with MS [6]. Besides, cognitive dysfunction is a major cause of disability in patients with MS [7]. Patients with MS, with cognitive dysfunction, often suffer from defects in the cognitive domains of memory, learning, attention, and information processing [8]. The concept of working memory has been evolved from short-term memory, with the difference that the latter stores information temporarily, and the information is not dependent on the structure of long-term knowledge.

In contrast, active or working memory is a multi-component system that performs the temporary storage and processing of information simultaneously [9, 10]. According to Baddeley, the concept of working memory refers to “a brain system that temporarily stores and manipulates information necessary for complex cognitive tasks, such as language comprehension, learning, and reasoning” [11]. The prevalence of cognitive dysfunction is estimated at 45–65%. In adults with MS, cognitive dysfunction is considered an adverse prognostic factor in the early stages of the disease [12]. In particular, studies show that cognitive impairment in these patients leads to unemployment and social and family constraints [13].

There has been no definite cure for MS until now. Treatment mainly consists of immunosuppressive and immune-modulating agents. However, several disease-modifying treatments (DMTs), such as interferon-beta (IFNβ) and glatiramer acetate (GA), have been designed to reduce the attack rate and delay disease progress [14]. One of the most significant parts of treatment is managing the different associated symptoms. [15]. Many MS patients hope that complementary and alternative medicine (CAM) will improve their quality of life. Patients dissatisfied with conventional therapies are more likely to use CAM [16]. In addition, the use of complementary medicine is on the rise in most parts of the world [17]. Research shows that over half of patients with MS use a variety of complementary or alternative therapies [18].

Complementary medicine, a low-risk, cost-effective, and easy therapeutic method, has trivial side effects, with aromatherapy being one of its branches [19]. Aromatherapy is an effective treatment in managing behavioral and psychological symptoms of dementia, which improves cognitive function, increases the quality of life, and enhances independence in daily life activities [20]. The study results by Jimbo *et al.* showed that aromatherapy was an effective non-drug therapy for people with dementia, which could have some potential for improving cognitive function, especially in patients with Alzheimer’s disease [21].

Lavender oil is obtained through steam distillation from the flowers of Lavandula angustifolia. Linalool and linalyl acetate are the main elements of this type of oil used in aromatherapy [22]. Linalool is a competitive antagonist of NMDA (N-Methyl D-Aspartic Acid) receptors, playing a key role in learning and memory processes [23]. However, little evidence has shown that aromatherapy with lavender essential oil affects the autonomic nervous system [24]. There are some studies on the effects of lavender essential oil on memory. Moss *et al.* conducted a study to evaluate the olfactory effects of lavender and rosemary essential oils on cognitive performance and mood in healthy volunteers. Accordingly, this study showed that inhalation of these essential oils had objective and subjective effects on cognitive performance and mood [25]. A study by Wattanathorn *et al.* showed that orange and lavender essential oils affected the working memory of healthy youths [26]. Various studies have been conducted on aromatherapy with lavender essential oil, yet different results have been reported. The study results by Bagheri *et al.* showed that lavender essential oil was ineffective in causing fatigue in hemodialysis patients [27]. However, in the study of Karadig *et al.* on ICU patients, sleep quality enhanced, yet their anxiety decreased [28]. In the study by Lin *et al.*, it improved the behaviors of people with dementia [29]. On the other hand, the study of Olapour *et al.* showed that pain decreased after a cesarean section [30]. The authors of the current research found no study to have specifically examined the effects of aromatherapy with lavender essential oil on working memory in MS patients. Thus, the present study was conducted to evaluate the effects of aromatherapy with lavender essential oil on working memory in women with MS who were referred to the MS Clinic in Rafsanjan in 2018.

## Material and Methods

This randomized clinical trial included 60 women with MS, referred to the MS clinic affiliated with Rafsanjan University of Medical Sciences for monthly checkups. The sample size, according to the degree of deviation of the study of Bahraini *et al.*, which was estimated at 11.96, upon considering α=0.05, β=10%, and d=12, was calculated at 21 according to the following formula. Due to the possibility of any sample dropout, 30 people were placed in each group [31].



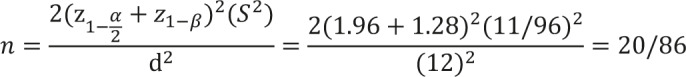



The samples were selected according to the inclusion criteria by a sampling method and were randomly assigned to the two intervention and placebo group comprising 30 individuals by drawing lots. The inclusion criteria were willingness to participate in the study, being within the age range of 20–40, having an education degree higher than a high school diploma, having an MS history of at least one year, having no history of undergoing aromatherapy during the last 6 months, lack of other chronic illnesses such as hypertension, lack of allergies, having no migraines, asthma, and other respiratory illnesses, and having a healthy sense of smell. Exclusion criteria were the increased severity of the disease in a way that increased the fatigue severity of the patient (increased severity of the disease that required visiting the doctor and changing the treatment process), hospitalization, acute illnesses, such as a fever, infections, cold, and severe pain, unwillingness to continue participation, exposure to acute or chronic stress, for example the death of a relative or unexpected accidents, pregnancies, and non-compliance with the consumption protocol.

Before the intervention, the patients’ sense of smell was tested with coffee. In the intervention group, in addition to routine treatments, the patients underwent aromatherapy with lavender essential oil twice a day for two weeks, each time for 10 minutes. Accordingly, the patients were trained to drip 2 drops of 100% lavender essential oil from Barij Essence Pharmaceutical Company of Kashan on a cotton ball using a dripper, placed at a 5 cm distance from their nose, and inhaled for 10 minutes. In the same vein, distilled water was used as a placebo in the placebo group [32]. A checklist and SMS texting were used to remind the patients of the intervention. Accordingly, a checklist was prepared to ensure the use of essential oils in the intervention group and distilled water in the placebo group, which was given to the patients and received from them in the end. Besides, SMS texting was used to remind the patients to perform the intervention twice a day. In addition, the questionnaire was completed before and after the last day of the intervention in the morning shift of the MS clinic.

In this study, data collection tools included a demographic information form (age, gender, employment status, marital status, MS type, history of using complementary medicine, and length of hospital stay) and the working memory test developed by Daneman and Carpenter [33]. The working memory test consisted of 27 sentences. Accordingly, the sentences were divided into six parts, a two-sentence part, a three-sentence part, a four-sentence part, a five-sentence part, a six-sentence part, and a seven-sentence part. This test was performed in 3 stages in this study. To this end, it was performed so that each part of the test was read for the subjects by starting from the two-sentence part to the seven-sentence part, respectively. In the meantime, they were asked to listen to these sections, each containing relatively difficult and unrelated sentences. In the end, they were asked to determine whether the sentences were semantically correct and to write down the last word of each sentence.

The first part measured the amount of the information processed, and the second part measured the amount of the information stored. The number of correct answers in each section was divided by the total number of sentences to score the working memory test. Next, the numbers obtained for each section were added to each other, divided by two, and then multiplied by 100. The final number obtained showed the amount of the working memory capacity of each subject.

The correlation of the working memory test developed by Daneman and Carpenter [33] was 0.59 for the verbal aptitude test, being 0.72 and 0.90 for the tests of real questions and pronouns, respectively [33]. Asadzadeh reported its reliability at 0.85 [34]. The collected data were analyzed by SPSS Statistics version 18.0 and the chi-squared test, Fisher’s exact test, Kolmogorov-Smirnov test, paired t-test, and independent t-test. The results were considered significant at the P<0.05.

## Results

According to the chi-squared test, there were no significant differences between the two studied groups in the marital status, disease duration, and length of hospital stay (P=0.739, P=0.301, P=0.481, respectively). Besides, based on Fisher’s exact test, there were no significant statistical differences between the two studied groups in the MS type, employment status, and history of taking complementary medicine (P=0.706, P=0.731, P=0.671, respectively). In addition, according to the independent t-test, there were no significant differences between the two groups in age (P=0.871). The two studied groups were homogeneous in terms of demographic characteristics (Table 1). According to the independent t-test results, there were no significant differences between the mean and standard deviation of working memory scores in the intervention and control groups before the intervention (P=0.304). However, the mean scores of working memories were significantly different (P=0.02) between the intervention and placebo groups after the intervention. According to intragroup comparison results and based on the paired t-test, the mean score of working memory before the intervention in the intervention group was 82.77±6.87, which increased to 87.64±5.57 after the intervention (P<0. 001). However, the placebo group had the average working memory scores of 80.30±11.09 and 82.09±11.31 before and after the intervention, respectively, which did not have a significant difference (P=0.154) (Table 2).

**Table 1.** Comparison of demographic characteristics in the two studied groups.

**Demographic Characteristics**	**Intervention Group**	**Placebo group**	**P-value**
**Average age (by year)**	33.20±6.97	33.46±5.56	P=0.871
**Duration of the disease**			
1–5 years More than 5 years	14 (43.8) 16 (57.1)	18 (56.3) 12 (42.9)	P=0.301
**Marital status**			
Single Married	5 (45.5) 25 (51.0)	6 (54.5) 24 (49.0)	P=0.739
**Employment status**			
Housewife Employed	26 (52) 4 (40)	24 (48) 6 (60)	P=0.731
**History of complementary medicine use**			
Yes No	4 (66.7) 26 (48.1)	2 (33.3) 28 (51.9)	P=0.671
**MS type**			
Relapsing-Remitting Other types	25 (48.1) 5 (62.5)	27 (51.9) 3 (37.5)	P=0.706
**Duration of hospitalization**			
Less than 1 year 1–5 years More than 5 years	16 (53.3) 12 (52.2) 2 (28.6)	14 (46.7) 11 (47.8) 5 (71.4)	P=0.481

**Table 2.** Comparison of the mean and standard deviation of working memory scores in patients in the two studied groups before and after the intervention.

**Working Memory Groups**	**Before the Intervention**	**After the Intervention**	**Paired T-test Result**
**Intervention Group**	82.77±6.87	87.64±5.57	P<0.001
**Placebo group**	80.30±11.09	82.09±11.31	P=0.154
**Independent T-test Result**	P=0.304	P=0.02	
Levene’s test of equality of error variances; dependent variable (after the intervention)	**F**=0.925 **df1**=2 **df2**=58 **Sig.**= 0.431		

## Discussion

This study was conducted to examine the effect of aromatherapy with lavender essential oil on the working memory of women with multiple sclerosis. Although memory impairment is one of the most common problems in patients with multiple sclerosis and the effects of lavender essential oil on memory have been confirmed in some studies, no study was found to examine the effects of lavender aromatherapy on the working memory of women with MS. Thus, it is difficult to evaluate the effectiveness of treatments due to the presence of conflicting reports on complementary medicine in studies with different designs and methods. However, this does not mean that complementary medicine does not affect MS. Due to the increased use of therapies in various diseases, including MS, more clinical trials are required in this regard [35]. The intragroup comparison results showed a significant statistical difference between the mean and standard deviation of the working memory score before and after the intervention. In other words, the results showed that aromatherapy with lavender essential oil led to a significant difference in the working memory function of women with MS (p<0.05).

Consistent with the findings of the present study, Fernandez’s study reported a positive effect of aromatherapy for lavender essential oil on nursing students’ memory [36]. In addition, the results of Wattanathorn’s study showed a significant difference in the mean score of the working memory of healthy youths in the two groups, i.e., aromatherapy with lavender essential oil and aromatherapy with orange essential oil [26]. The study by Jimbo *et al.* examined the effects of aromatherapy using rosemary and lemon essential oils in the morning as well as lavender and orange essential oils at night in people with dementia; accordingly, they showed that aromatherapy was an effective non-drug therapy in people suffering from the disease [21].

Positive effects of aromatherapy with lavender essential oil were reported in these studies. However, there were differences in the research community and type of aroma, which included lavender in the present study and a combined aroma in most other studies. This could be indicative of the effect of lavender on memory. The similar results achieved keep alive the hope for preventing and treating dementia and memory impairment using medicinal plants in the future. Accordingly, further studies are needed in this field. Inconsistent with the findings of the present study, the results of Moss *et al.* showed that lavender reduced functions of working memory and secondary memory. Besides, it impaired memory speed and attention to key tasks, compared to the control group [25]. Inconsistent results of the mentioned study with those of the present one could be due to the differences in the research community, tools for measuring working memory, as well as frequency and duration of aromatherapy use.

One of the strengths of this study compared to other ones was the use of lavender essential oil alone, not in combination. On the other hand, one of the limitations of this study could be the possibility of the patients not following the consumption protocol at home. Using SMS texting and checklists, we tried to remove this limitation. In addition, if a patient did not undergo aromatherapy, they would be dropped out of the study.

## Conclusion

According to the present study results, inhalation aromatherapy with lavender essential oil significantly improved working memory among MS patients. Furthermore, due to the growing use of complementary medicine among patients with MS, it seems possible to use this method and teach them how to use it if they are willing.

## Acknowledgments

### Conflict of interest

The authors declare that there is no conflict of interest.

### Ethical approval

This study was approved by Rafsanjan University of Medical Sciences with code IRCT20170122032111N4 received from the Iranian Registry of Clinical Trials (IRCT) and ethics code IR.RUMS.REC.1397.047.

### Consent to participate

Written informed consent was obtained from the participants in this study.

### Funding

This article was funded by Rafsanjan University of Medical Sciences.

### Personal thanks

The researchers would like to thank all participants for cordially assisting us in doing this research. Furthermore, the researchers would like to thank all staff members of the Special Diseases Department at Ali ibn Abi Talib hospital and the Rafsanjan University of Medical sciences.

### Authorship

SMR and MS contributed to conceptualizing the study, writing the original draft. ARS and ML contributed to the methodology. ML contributed to editing the manuscript. SMR contributed to data collection and ARS to data curation and analysis.
